# Case series: 3D printed orthopedic brace combined with traditional manipulative physiotherapy to treat new-onset scoliosis in adults

**DOI:** 10.1097/MD.0000000000028429

**Published:** 2022-01-07

**Authors:** Hui Jin, Ziyu Zhang, Yao Gao, Huan He, Shibin Feng, Rui Xu, Qiang Li, Hao Zuo

**Affiliations:** a Department of Pain, The Second Hospital of Jilin University, Changchun, Jilin, PR China; b Norman Bethune Health Science Center of Jilin University, Changchun, Jilin, PR China; c Department of Endocrinology, Shanghai National Research Center for Endocrine and Metabolic Disease, State Key Laboratory of Medical Genomics, Shanghai Institute for Endocrine and Meta-bolic Disease, Ruijin Hospital, Shanghai Jiaotong University School of Medicine, Shanghai, PR China; d Department of Orthopedics, Jilin Provincial People's Hospital, Changchun, Jilin, PR China.

**Keywords:** 3D printing, Cobb angle, orthopedic braces, scoliosis, traditional manipulative treatment

## Abstract

**Introduction:**

New-onset scoliosis in adults is different from that in congenital and idiopathic scoliosis. We applied personalized custom 3D printed orthopedic braces combined with traditional manipulative physiotherapy to treat adult patients with new-onset scoliosis and observed the effectiveness of the treatment.

**Patient concerns:**

Nine patients aged 20–52 years presented with unequal height of hips and asymmetrical waist. One shoulder was obviously protruding or enlarged compared to the other; when lying on the bed, the legs were not equal in length, and when bending down, the back was not equal on the left and right.

**Diagnosis:**

New-onset scoliosis.

**Interventions:**

Application of individual customized 3D printing brace combined with traditional treatment. Evaluation of clinical efficacy after treatment, including functional exercise test (FMS) before and after treatment, ability of daily living (ADL), visual analog pain score (VAS), and scoliosis angle (Cobb angle).

**Outcomes:**

The total effective treatment rate was 100.00% (9/9). The VAS score, Cobb angle of the spine, FMS test, and ADL test were significantly improved compared with those before treatment.

**Conclusion:**

The customized 3D printed orthopedic brace combined with traditional techniques to treat scoliosis and innovatively combined human bionic technology with traditional medicine to achieve the continuity and precise correction of scoliosis treatment is a clinically effective technique.

## 1 Introduction

Scoliosis refers to a spinal deformity in which one or several segments of the spine are bent sideways or accompanied by rotation of the vertebral body.^[1]^ Clinically, it is divided into congenital, idiopathic, and neuromuscular origin, traumatic degeneration, functional, degenerative scoliosis, etc.^[2]^ Except for congenital and idiopathic scoliosis, which are more common in adolescents, the rest are more common in adulthood, which scholars call adult new-onset scoliosis.^[3]^ New-onset scoliosis in adults is different from that in congenital and idiopathic scoliosis. In recent years, with the development of society, the pace of life has accelerated, and the number of people with poor sub-health and long-term behavior patterns has increased. The incidence of the disease has been increasing annually, and is not uncommon in clinical practice.^[4]^ Adult new-type scoliosis usually has the following characteristics: ① it usually occurs in adulthood, the spine develops normally, the bones are mature, and the vertebral body is not damaged or destroyed; ② patients usually go to the doctor with chest, back, or lumbar pain, usually accompanied by long-term behavior Bad medical history; ③ The scoliosis is a compensatory change, and the position of the scoliosis is related to the painful location. The pathological basis is the imbalance of muscle strength on both sides of the spine; ④ The imaging manifestations include coronal scoliosis and sagittal physiological curvature change.^[5]^

In terms of treatment methods, patients with a scoliosis angle (Cobb angle) of < 40° account for the majority, and conservative treatment is the main method to correct three-dimensional compensation and improve muscle imbalance at the same time.^[6]^ Currently, the recognized method for adult new-onset scoliosis is to use conventional orthopedic braces and/or combined with rehabilitation treatment methods such as traction, acupuncture, and exercise therapy, but it generally has a long cycle, patients cannot tolerate the treatment, and multi-dimensional correction is difficult.^[2]^ Current brace treatment is mainly for coronal scoliosis, and the curative effect is poor. Treatment of axial and sagittal deformities is very rare, and such braces are even more difficult to observe.^[7]^ This research innovatively uses 3D a personalized custom orthopaedic brace to simulate the treatment of a therapist, 1:1 copy the shape of the patient's hand, and printing it to form a wearable brace, which is highly targeted for scoliosis lesions and has accurate correction; combining traditional treatments to relieve the discomfort of patients and increase the continuity of treatment, it is suitable for conservative treatment of patients with scoliosis, and the effect is satisfactory.

## 2 Methods

From July to August 2020, the Pain Department of the Second Hospital of Jilin University admitted nine patients with scoliosis as research subjects. All patients provided informed consent for participation and publication of the case details for research purposes. The criteria for admission were as follows: ① age over 18 years old; ② the spine presents simple scoliosis, and the line of vertebral spinous processes deviates from the central axis; ③ the muscles on both sides of the spine are asymmetrical, usually accompanied by abnormal skin temperature on the concave side; numbness, paralysis, and pain symptoms; ④ shoulders, scapula, and iliac crests of the pelvis are not equal in height; ⑤ Adam flexion test is positive; ⑥ Imaging examination: Erectile X-ray anterior spine film shows unilateral curvature, Cobb angle 10°–40°, lesions include chest section, thoracic waist section, and lower waist section. At least one of the above items (②–⑥) is a positive sign.

### 2.1 Interventions

#### 2.1.1 Customized 3D printed orthopedic brace

**Figure 1:**
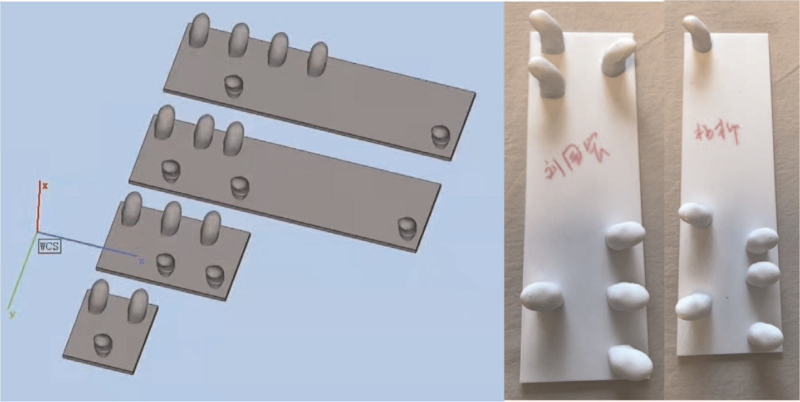
Modeling and printing of customized 3D printing brace.

**Figure 2:**
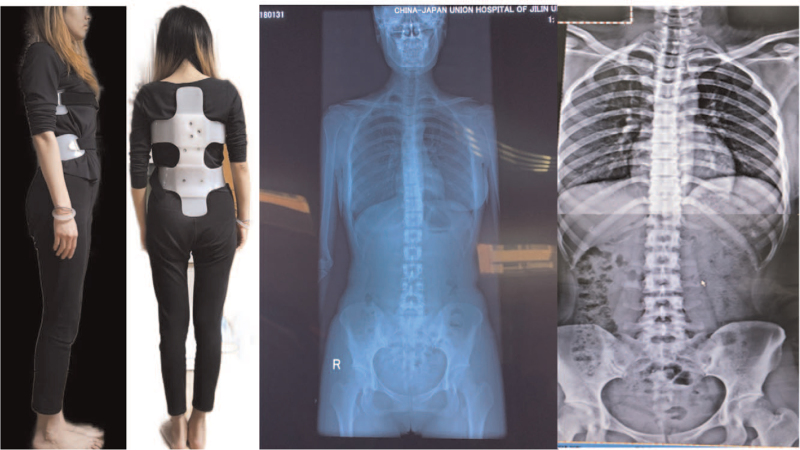
Treatment and comparison of Cobb angle before and after treatment.

Before starting treatment for each patient, the FMS, ADL, VAS, and other scores were tested and spinal X-rays were taken. According to the position of each patient's scoliosis and the rotation of the axial plane, the brace was personalized, and the part of the brace supporting the spine was designed according to the size of the patient's thumb, and EinScanPro was used to collect data and model; 1:1 copy the simulated therapist's treatment hand shape and print it to form a wearable brace, input the data into the Fire2plus 3D printer, and ABS resin was used to print the finished brace (Fig. 1). The radiographs of the spine were reviewed after each patient was treated with a brace (Fig. 2). After 1 h of treatment, the patient removed the brace and performed traditional treatment to relieve discomfort.

#### 2.1.2 Traditional manipulative physiotherapy

The patient is in the prone position, strokes the spine from bottom to top, and rubs the palm roots on the concave side of the foot sun bladder meridian, foot Yangming stomach meridian, and foot Shaoyang gall bladder meridian. This means pressing and kneading to relax the left erector spinae, left lumbar muscles, and bilateral hamstrings. Under your hands, you can feel that you can pluck the nodules, then fix the right lumbar vertebrae at the base of the palm and push the vertebrae from the right to the left. Relax and stretch the left quadratus lumbar muscle in the right decubitus position. The patient's iliopsoas and rectus femoris were relaxed in the supine position. Ask the patient to perform abdominal crunching exercises, 20 in each group, and 3 groups. Train the right hip muscles in the left decubitus position, with elastic bands to resist resistance, 20 per group, 3 groups. The standing position allows the patient to perform the right side flexion exercise, and the right hand can take the water bag to increase the strength. There were 20 groups in each group and 3 groups. For each group of training, the patient can feel the muscle soreness, not soreness, and the stretching of the muscles can also increase the strength one by one with the strength that the patient can withstand. The overall treatment idea is to relax and stretch the concave side muscles and train the convex side muscles. During the entire treatment cycle, the convex-side muscles can be stimulated with an intermediate-frequency pulse electric physiotherapy instrument.

### 2.2 Efficacy evaluation criteria

The functional exercise test (FMS), the ability of daily living (ADL), the visual analog pain score (VAS), and the changes in the Cobb angle before and after treatment were recorded in nine patients. ①FMS functional movement assessment, namely squat, hurdles, straight lunges, shoulder flexibility, supine straight leg lifts, trunk stabilization push-ups, trunk rotation stability, 7 basic movements and three exclusion movements to perform the body flexibility, body balance, body coordination ability and neuromuscular response ability are a method of testing and scoring assessment. It is mostly used for human exercise ability tests.^[8]^②Activity of daily living (ADL) refers to the necessary activities and functional movements that an individual performs daily to meet the needs of daily life. It is often used to evaluate the quality of life of patients’ activities of daily living before and after rehabilitation,^[9]^ and ③ pain assessment. Pain is often the main reason for patients to see a doctor, and it is also the main appeal that is eager to solve. The visual analog scale (VAS) is the most commonly used method for evaluating pain.^[10]^④Observation of Cobb angle: Before and after treatment, patients were taken to take radiographs of the spine in the standing position, and the Cobb angles of scoliosis before and after treatment were measured (the upper and lower end vertebrae of each measurement were consistent to prevent measurement errors). Cure: Physiological curvature is normal, scoliosis deformity basically disappears, Cobb angle <5°; marked effect: physiological curvature is basically normal, scoliosis deformity is significantly improved, Cobb angle <10°; improvement: scoliosis deformity is improved, Cobb angle improvement degree ≥5°; Invalid: Cobb angle improvement degree <5°. Total effective rate = cure rate + apparent rate + improvement rate.

## 3 Results

The chief complaints of the patients, including the hips, were not equal in height, and the waist was asymmetrical. One shoulder was obviously protruding or enlarged. When lying on the bed, the legs were not equal in length. When bending down, the back was not equal to the left and right sides.

There were 1 male and 8 female subjects. Their ages ranged from 20 to 52 years, with an average age of 31.7 years old. Lesions: 3 cases of thoracic and lumbar segments, 8 cases of thoracic segments, and 7 cases of lower lumbar segments Some patients had multiple segmental lesions.

According to the comparison of the FMS scale score distribution before and after treatment, the scores of each index have a rising trend, suggesting that the patient's motor function has improved significantly; while comparing the distribution of ADL scale scores before and after treatment, some patients’ scores tended to increase, but the increase was not obvious. The main reason is that the daily behavioral activities of such patients are not significantly restricted; Comparing the distribution of VAS scores before and after treatment, the scores of all indicators have a decreasing trend, suggesting that the pain of patients decreases after treatment; Before and after treatment, the Cobb angle of the spine tended to decrease. According to the above-mentioned Cobb angle observation index, the patients in this study reached the improvement or above requirements, so the overall effective rate was 100% (Table 1).

**Table 1:** Comparison of observation indexes of 9 subjects before and after treatment.

			Cobb angle		VAS		FMS		ADL	
Patient	SEX	AGE	Before	After	Before	After	Before	After	Before	After
1	Female	20	10.2	3.8	3	2	11	12	100	100
2	Female	20	12.4	2.7	4	2	11	13	100	100
3	Female	27	10.8	0	3	1	12	14	100	100
4	Female	35	11.1	1.9	0	0	5	8	80	100
5	Female	25	10.7	3.8	5	1	9	12	100	100
6	Female	45	11.1	3.9	0	0	1	5	35	100
7	Female	52	13.6	4.8	0	0	2	5	60	90
8	Male	20	10	2.7	3	2	12	14	100	100
9	Female	42	10.7	1.8	3	1	3	6	90	100

## 4 Discussion

At present, in the clinical treatment of scoliosis, conservative treatment should be adopted as far as possible when the Cobb angle is less than 40°.^[11]^ In the clinical conservative treatment of scoliosis, rehabilitation treatment (including traditional Chinese medicine treatment and modern rehabilitation treatment technology) and brace treatment are the main methods.^[12]^ Traditional Chinese medical manipulative treatments include acupuncture, massage, orthopedics, chiropractic, and acupotomy.^[13]^ The treatment of scoliosis is clinically effective, easy to operate, and inexpensive, but its treatment dimensions are often relatively single, so long-term efficacy is often poor. Modern rehabilitation treatments include exercise therapy, core muscle training, suspension, transcutaneous electrical stimulation, etc.^[14]^ Patients need a longer period of rehabilitation treatment, and often fail to persist in treatment for a long time, leading to treatment failure; ②The brace treatment of scoliosis is one of the effective clinical methods.^[12]^ Patient compliance with brace treatment was the main factor affecting the treatment effect. The patient needs to wear it for a long time, which causes physical pain to the patient.^[15]^ In addition, traditional brace treatment mainly performs deformity correction from the coronal plane, and the accuracy needs to be further improved.^[16]^ The above treatment methods mainly focus on coronal scoliosis, while coronal and sagittal scoliosis occurs because of the rotation of the horizontal plane.^[17]^ Therefore, the current treatment is mostly of a single dimension.

This study innovatively applies individualized customized 3D printing technology for scoliosis correction. This study has two innovations. ①The brace made by 3D printing technology was used in this study, customized and modeled according to the shape of each patient's treatment hand, 1:1 restored the angle and location of the therapist's manual treatment, precise treatment for the lesion area, and treatment time of one hour.^[18]^②The 3D printing brace in this study has the ability to perform three-dimensional correction for the rotation deformity of the axial plane and the deformity of the sagittal plane and the horizontal plane.^[19]^ The 3D printed orthopedic brace realized the reduction therapist's technique at the same time and realized the rotation and sagittal deformity of the axial plane simultaneously.^[20]^ It is an upgrade to the treatment concept of the disease and has achieved satisfactory clinical effects. In this study, the FMS scores and Cobb angle of 9 patients changed significantly before and after treatment, suggesting that the patients had significant improvement in motor function test and scoliosis angle after treatment. This shows that the 3D printed orthopedic brace is effective in treating adult new-onset scoliosis, and the VAS score and ADL score were also partially improved. This shows that the combination of manual treatment alleviates the patient's discomfort, improves comfort, and contributes to the continuity of treatment. There are still many limitations to our study. There is still room for improvement in the degree of brace customization. The use of composite materials and new materials can improve patient comfort. In the future, we will expand the number of subjects to obtain more credible results.

In summary, individualized 3D printed brace combined with traditional techniques are satisfactory for the treatment of scoliosis. The innovative individualized 3D printed brace can provide multi-dimensional and precise treatment, and effectively alleviate patient discomfort to ensure the continuation of treatment. This is the next innovative breakthrough in the trend of precision treatment, and it is worthy of popularization and application.

## Acknowledgments

None.

## Author contributions

**Conceptualization:** Hui Jin, Ziyu Zhang, Yao Gao, Huan He, Shibin Feng, Rui Xu, Qiang Li, Hao Zuo.

**Data curation:** Hui Jin, Ziyu Zhang, Yao Gao, Huan He, Shibin Feng, Rui Xu, Qiang Li, Hao Zuo.

**Formal analysis:** Hui Jin, Ziyu Zhang, Yao Gao, Huan He, Shibin Feng, Rui Xu, Qiang Li, Hao Zuo.

**Funding acquisition:** Hui Jin, Ziyu Zhang, Yao Gao, Huan He, Shibin Feng, Rui Xu, Qiang Li, Hao Zuo.

**Investigation:** Hui Jin, Ziyu Zhang, Yao Gao, Huan He, Shibin Feng, Rui Xu, Qiang Li, Hao Zuo.

**Methodology:** Hui Jin, Ziyu Zhang, Yao Gao, Huan He, Shibin Feng, Rui Xu, Qiang Li, Hao Zuo.

**Project administration:** Hui Jin, Ziyu Zhang, Yao Gao, Huan He, Shibin Feng, Rui Xu, Qiang Li, Hao Zuo.

**Resources:** Hui Jin, Ziyu Zhang, Yao Gao, Huan He, Shibin Feng, Rui Xu, Qiang Li, Hao Zuo.

**Software:** Hui Jin, Ziyu Zhang, Yao Gao, Huan He, Shibin Feng, Rui Xu, Qiang Li, Hao Zuo.

**Supervision:** Hui Jin, Ziyu Zhang, Yao Gao, Huan He, Shibin Feng, Rui Xu, Qiang Li, Hao Zuo.

**Validation:** Hui Jin, Ziyu Zhang, Yao Gao, Huan He, Shibin Feng, Rui Xu, Qiang Li, Hao Zuo.

**Visualization:** Hui Jin, Ziyu Zhang, Yao Gao, Huan He, Shibin Feng, Rui Xu, Qiang Li, Hao Zuo.

**Writing – original draft:** Hui Jin, Ziyu Zhang, Yao Gao, Huan He, Shibin Feng, Rui Xu, Qiang Li, Hao Zuo.

**Writing – review & editing:** Hui Jin, Ziyu Zhang, Yao Gao, Huan He, Shibin Feng, Rui Xu, Qiang Li, Hao Zuo.
